# A Wholesale Cleaner

**Published:** 1912-01-15

**Authors:** 


### A WHOLESALE CLEANER.

The following is taken from a recent issue of the Saturday Evening Post and has to do with Dr. Oscar Dowling, Health Officer of the State of Louisiana who was elected one of the Directors of the recently formed National Mouth Hygiene Association. We have mentioned in a previous issue, the fact that the exhibit of the Rochester Dental Society was a part of the display of this "Health Train."—DR. W. W. BELCHER, Editor.
Dental Dispensary.

Do you remember—of course you do—that good old one about the surveying party that was working in Arkansas and came to the cabin of a farmer? It was a hot day and they asked the farmer for something to drink. The farmer had buttermilk and brought round a pitcher of it.

"This would be great," said one of the thirsty surveyors, "if we had some ice to put in it. Say, my friend, have you any ice?"

"Ice!" snorted the farmer. Who in blazes ever heard of ice in the summer time!"

Same thing happened in Alabama long before. There was a church row over the proposition in the town where Oscar Dowling lived. Some of the brethren allowed there could be ice in the summer and there should be a few chunks of it at the church picnic. Others claimed ice in the summer was against Nature. It couldn't happen! Things looked squally for the picnic and for the church until Oscar Dowling took hold. A habit he has, that Dowling, of taking hold of things. He got a block of ice and took it to the picnic, and the proceedings were marked with peace and amity—and ice.

He used to edit a newspaper. It wasn't much of a newspaper, but he was a whale of an editor. One day he went to the man who owned the paper and said:

"Say, why don't you boost the town with that paper of yours?"

"Huh!" retorted the owner. "I like your nerve! If you want to boost the town with the paper buy the paper and do your own boosting."

Wherefore Oscar bought the paper and proceeded to boost. He thought it would be a good plan to print pictures of the local celebrities. The first person he pictured was the minister. The community was shocked. Fancy it! There was the picture of the minister right in the paper! The minister, being alive to the advantages of advertising, even for his calling, didn't object and the community soon recovered from the shock, especially as Oscar printed the pictures of a few of the most shocked and showed them it wasn't so unpleasant to have the neighbors say: "Saw your picture in the paper today."

Also, Oscar introduced the first press dispatches in that section of the country. They came in by train, and Jim, the devil, brought them up to the office by mustang. This was too slow, so Oscar rigged up a cigar-box telephone from the station to the drug store; and when the train came in Jim telephoned the news up to the store and Oscar took it hot off the string and put it in the paper.

There were lots of other things in the town that didn't suit Oscar. He criticised the methods of the man who ran the livery stable. "Dodgast it!" said the man; "I've bin runnin' this stable for twenty years. If you don't like it, buy it and run it yourself!" Oscar bought it and ran it. Also, he bought the hotel and the drug store on the principle that they were not run right either, and proceeded to demonstrate his theory of editing, innkeeping, drug-storing and livery-stable keeping. There is no telling how much farther he would have gone in reconstructing that town if his mother hadn't decided he must be a doctor.

### HOW A STATE GOT ITS FACE WASHED.

Oscar was willing, but being a doctor with him meant being a real doctor; so he studied medicine at Vanderbilt University and then took courses of from one to three years at clinics in New York, Chicago, New Orleans, London, Berlin, and shorter courses in Paris and the City of Mexico. Once, when a patient of his died in a country town and there was no hearse, he borrowed a hearse from a neighboring village. Nobody would drive the hearse. That according to local belief, would bring bad luck. Therefore Oscar drove the hearse himself—and drove it well.

It wasn't long before Oscar was one of the great doctors of Louisiana. He specialized as an oculist. The medical profession recognized him as a leader, and he was made president of all their organizations. Having been an editor in his youth he kept on writing for medical publications. Then in September, 1910, he was made president of the State Board of Health of Louisiana.

He was giving up a large practice for a salary of a hundred dollars a week, but he had ideas about sanitation, and he took the place—grabbed it, in fact. Then he removed his coat, rolled up his shirt-sleeves and announced:

"We shall now proceed to clean up the state."

"But how?" asked the citizens, aghast.

"By the simple expedient of removing the dirt," Oscar replied.

No sooner said than done. He planned a trip through Louisiana with a "health train." He laid the plan before the railroad men.

"Dowling," said one railroad president whom he had asked for a car, "you are a nice fellow—but a jackass!"

"Undoubtedly correct," smiled Oscar at the railroad president: "but give me that car!"

Instead of one car he secured three. Then began the remarkable trip through the state. It was last November. The health train had a car containing pictures and exhibits showing how disease can be prevented by the removal of dirt. The second car contained models of all sorts of sanitary appliances—from drains to labor-saving and dirt-preventing utilities for kitchens. The doctor and his assistants lived in the third car.

They covered the state, stopping at all sorts of villages and cities. They gave practical demonstrations of how towns can be cleaned, had moving-picture shows and lectures and inspections. The theory of Doctor Dowling was that the best possible health environment can be secured through local aid, and local aid can be enlisted by a revelation of conditions and the education of the public mind. Inspection was the most forcible feature of the work. When the train reached a village or city Doctor Dowling and his force of inspectors began work. They visited hotels, restaurants, bakeries, butcher shops, schools, outhouses, stores, public buildings and residences.

Oscar was everywhere. He worked like a steam engine and went through those kitchens and stores and other places like an avenger. Nothing escaped his eye. He saw grease on a stove, dirt in the milk cans, dough on kneading boards, as quickly as he detected unsanitary plumbing or other unhealthful conditions. It was all dirt to him, and to be eliminated. He didn't stand round and talk either. He demonstrated. When he thought the housekeeper needed a little practical instruction he showed her how to keep her kitchen clean by calling for soap and water and giving an object-lesson.

Well, the way he renovated the state of Louisiana was a caution! He would rush into a town, inspect it, tell the people just what meat market and what restaurants and what dairies where dirty; show how this dirt helped the spread of disease, and then order a general cleaning up. He made them clean up too! There was no bluffing about it. "Clean up or shut up!" said the doctor. They had to do it. As one marketman in a Louisiana town said in a handbill he distributed. "To my Customers: This is to notify you that I will discontinue my market after April thirtieth, until I can meet the requirements of the State Board of Health, which will be only a short time."

He visited more than two hundred cities, towns and villages, and cleaned them. He established a force of local inspectors that is keeping them clean. There was a good deal of protest. No community likes to be told it is living in filth. Still, Dowling went along and told all communities that was exactly what they were doing—and proved it. As one editor put it: "The more our people read and think about Doctor Dowling's scathing report of the unsanitary conditions existing in and around the town the madder we become; and if this thing keeps on we're likely to get mad enough to clean up!"

After Dowling had made his state campaign he took a train out into other states. Having scared Louisiana stiff and made that state clean up, the doctor traveled round and told the people of other states how much Louisiana had improved, how much it was to improve, and how that state had been lied about and traduced as to its health conditions. All of which shows him to be a good sport.

He is forty-four years old, with the heart of a boy and the winning smile of a woman. He is good-natured and happy, but with tremendous energy; and can be as stern as any circumstances demand. He has kept politics out of the State Board of Health and has secured wonderful results. He has proved himself a remarkable organizer and administrator, and the whole state believes in him.—*Saturday Evening Post.*

